# Three closely related 1-[(1,3-benzodioxol-5-yl)methyl]-4-(halobenzo­yl)piperazines: similar mol­ecular structures but different inter­molecular inter­actions

**DOI:** 10.1107/S2056989019000458

**Published:** 2019-01-11

**Authors:** Ninganayaka Mahesha, Belakavadi K. Sagar, Hemmige S. Yathirajan, Tetsundo Furuya, Tomoyuki Haraguchi, Takashiro Akitsu, Christopher Glidewell

**Affiliations:** a Department of Studies in Chemistry, University of Mysore, Manasagangotri, Mysuru-570 006, India; b Department of Chemistry, Faculty of Science, Tokyo University of Science, 1-3 Kagurazaka, Shinjuku-ku, Tokyo 162-8601, Japan; c School of Chemistry, University of St Andrews, St Andrews, Fife KY16 9ST, UK

**Keywords:** piperazines, crystal structure, mol­ecular conformation, hydrogen bonding, supra­molecular assembly

## Abstract

In each of the compounds 1-[(1,3-benzodioxol-5-yl)methyl]-4-(3-fluoro­benzo­yl)piperazine, C_19_H_19_FN_2_O_3_ (I), 1-[(1,3-benzodioxol-5-yl)methyl]-4-(2,6-di­fluoro­benzo­yl)piperazine, C_19_H_18_F_2_N_2_O_3_ (II), and 1-[(1,3-benzodioxol-5-yl)methyl]-4-(2,4-di­chloro­benzo­yl)piperazine, C_19_H_19_Cl_2_N_2_O_3_ (III), the piperazine rings adopt a chair conformation with the (1,3-benzodioxol-5-yl)methyl substituent occupying an equatorial site: the five-membered rings are all slightly folded across the O⋯O line leading to envelope conformations. The dihedral angle between the planar amidic fragment and the haloaryl ring is 62.97 (5)° in (I) but 77.72 (12)° and 75.50 (5)° in (II) and (III), respectively. Despite their similarity in constitution and conformation, the supra­molecular inter­actions in (I)–(III) differ: in (I), a combination of C—H⋯O and C—H⋯π(arene) hydrogen bonds links the mol­ecules into a three-dimensional framework structure, but there are no hydrogen bonds of any sort in either (II) or (III), although the structure of (III) contains a short Cl⋯Cl contact between inversion-related pairs of mol­ecules.

## Abstract 2

Three 1-[(1,3-benzodioxol-5-yl)methyl]-4-(halobenzo­yl)piperazines adopt very similar mol­ecular conformations but, while the mol­ecules of the 3-fluoro­benzoyl are linked by hydrogen bonds into a three-dimensional structure, there are no hydrogen bonds in either of the 2,6-di­fluoro­benzoyl and 2,4-di­chloro­benzoyl analogues.

## Chemical context   

**Figure 1:**
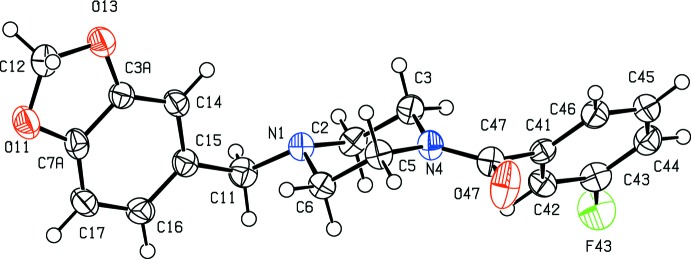
The mol­ecular structure of compound (I) showing the atom-labelling scheme. Displacement ellipsoids are drawn at the 50% probability level.

1-[(1,3-Benzodioxol-5-yl)meth­yl]piperazine is an important inter­mediate for the synthesis (Duncton *et al.*, 2006 ▸; Hamid & Williams, 2007 ▸) of piribedil, 1-[(1,3-benzodioxol-5-yl)meth­yl]-4-(pyrimidin-2-yl)piperazine, which is used in the treatment of Parkinson’s disease, particularly in the reduction of tremor (Rondot & Ziegler, 1992 ▸; Millan *et al.*, 2001 ▸). The synthetic routes to piribedil reported hitherto have utilized either palladium-catalysed (Duncton *et al.*, 2006 ▸) or ruthenium-catalysed (Hamid & Williams, 2007 ▸) processes, requiring extensive purification procedures to ensure that the final product is free of heavy metals. With this in mind, we have now synthesized a series of *N*-aroyl analogues (I)–(III) (Figs. 1 ▸–3 ▸ ▸) using a metal-free procedure involving a straightforward coupling reaction between 1-[(1,3-benzodioxol-5-yl)meth­yl]piperazine and a carb­oxy­lic acid, promoted by 1-(3-di­meth­yl­amino­prop­yl)-3-ethyl­carbodimide as the dehydrating agent, and we report here the mol­ecular and supra­molecular structures of compounds (I)–(III).
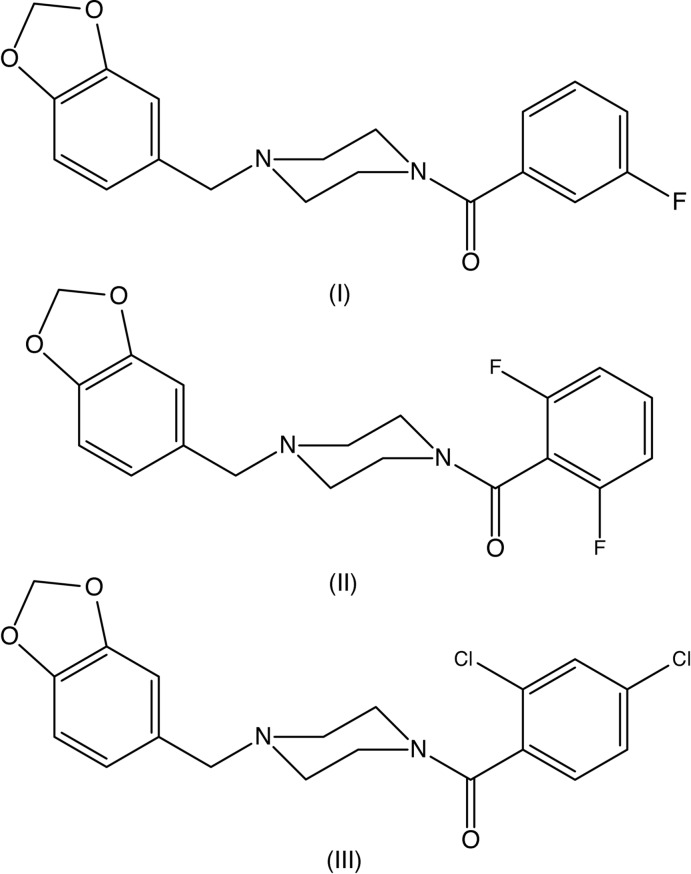


## Structural commentary   

In each of (I)–(III), the five-membered ring is slightly non-planar: while the atoms O11, C7*A*, C3*A* and O13 are co-planar, as expected, the atom C12 is slightly displaced from this plane by 0.150 (2), 0.099 (6) and 0.210 (2) Å in (I)–(III), respectively, giving an envelope conformation in each case, with the ring folded across the line O11⋯O13. The piperazine rings all adopt chair conformations with the substituent at atom N1 in an equatorial site, while the atoms of the amide fragment (C3, N4, C5, C47, O47 and C41) are coplanar. The only significant conformational difference between the mol­ecules in (I)–(III) lies in the dihedral angle between the amide unit and the adjacent aryl ring (C41–C46), 62.97 (5)° in (I) but 77.72 (12) and 75.50 (5)° in (II) and (III), respectively. The mol­ecules of (I)–(III) exhibit no inter­nal symmetry and hence they are all conformationally chiral, but the space groups (Table 2 ▸) confirm that equal numbers of the two conformational enanti­omorphs are present in each crystal.

## Supra­molecular features   

**Figure 4:**
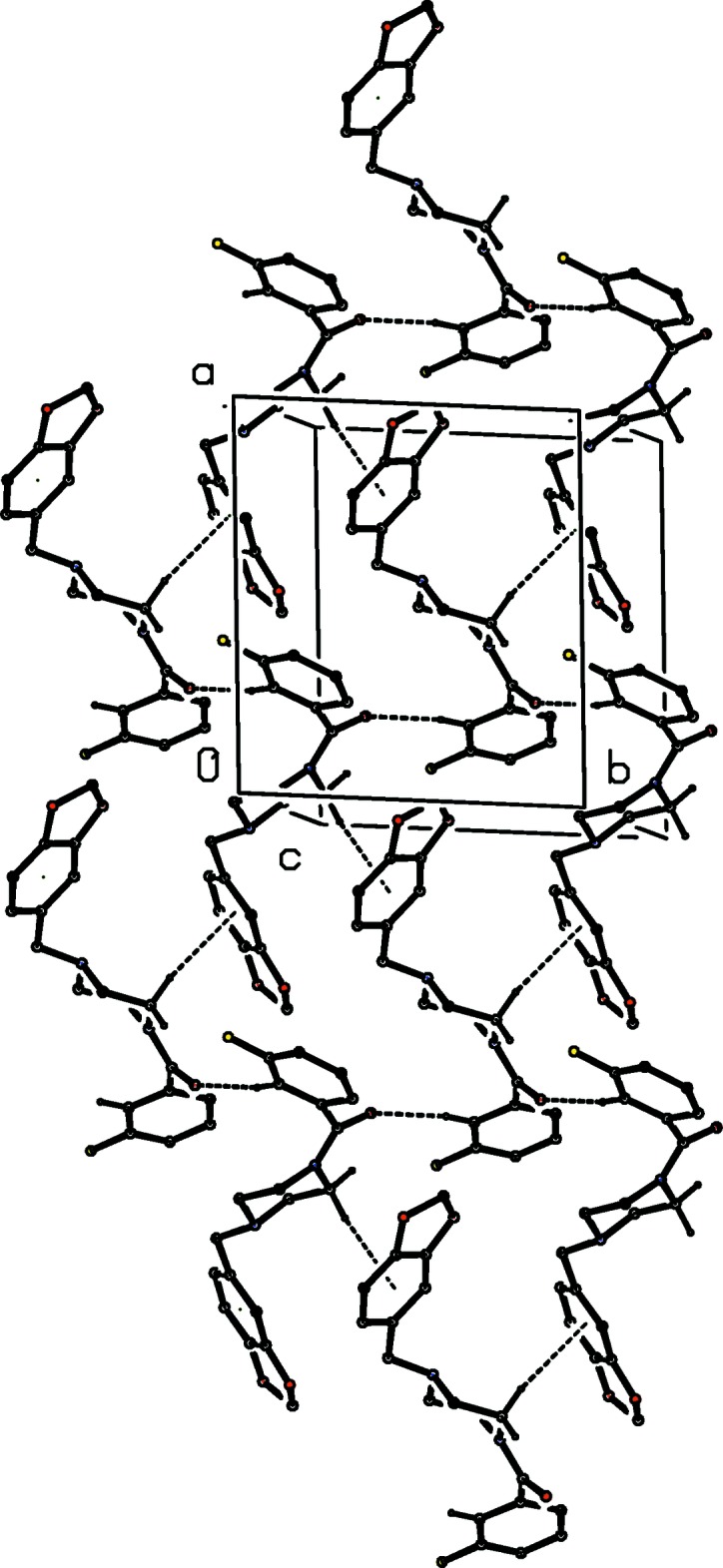
Part of the crystal structure of compound (I) showing the formation of a sheet lying parallel to (001) and built from C—H⋯O and C—H⋯π(arene) hydrogen bonds, which are drawn as dashed lines. For the sake of clarity, the H atoms bonded to the C atoms not involved in the motifs shown have been omitted.

**Figure 5:**
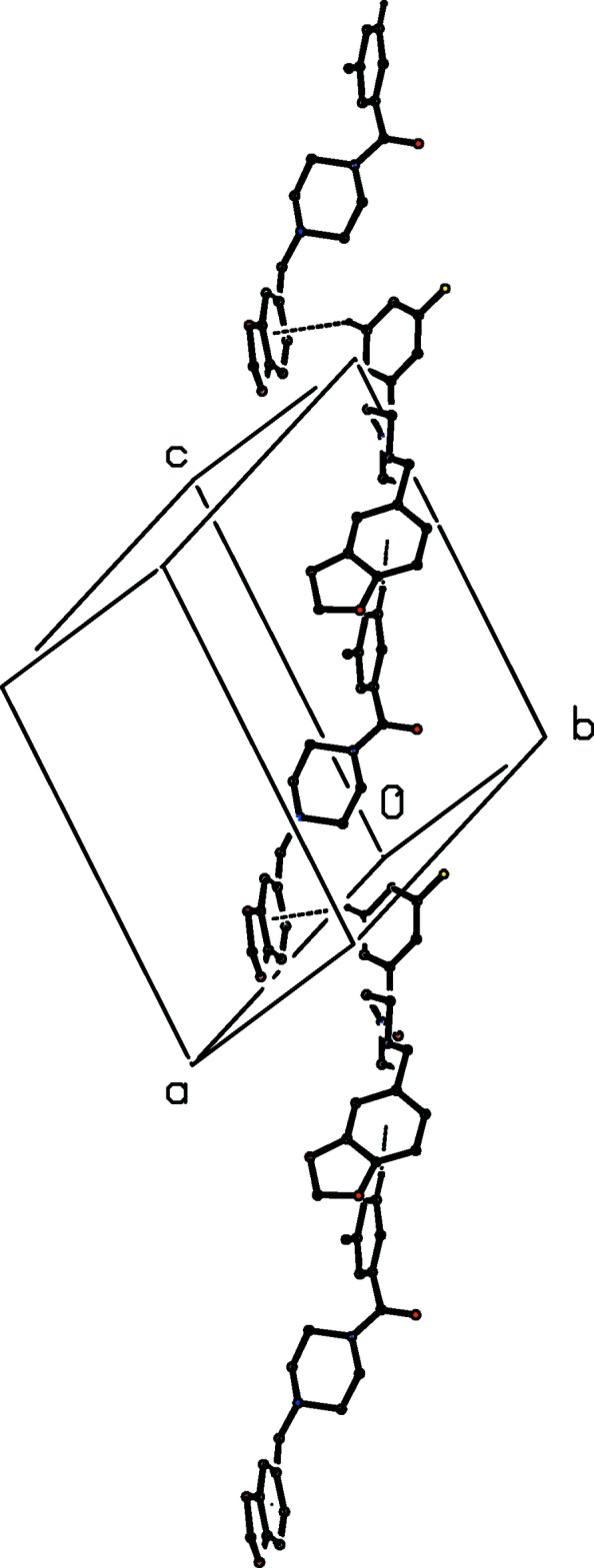
Part of the crystal structure of compound (I) showing the formation of a chain running parallel to [10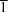] and built from C—H⋯π(arene) hydrogen bonds, which are drawn as dashed lines. For the sake of clarity, the H atoms not involved in the motifs shown have been omitted.

**Figure 6:**
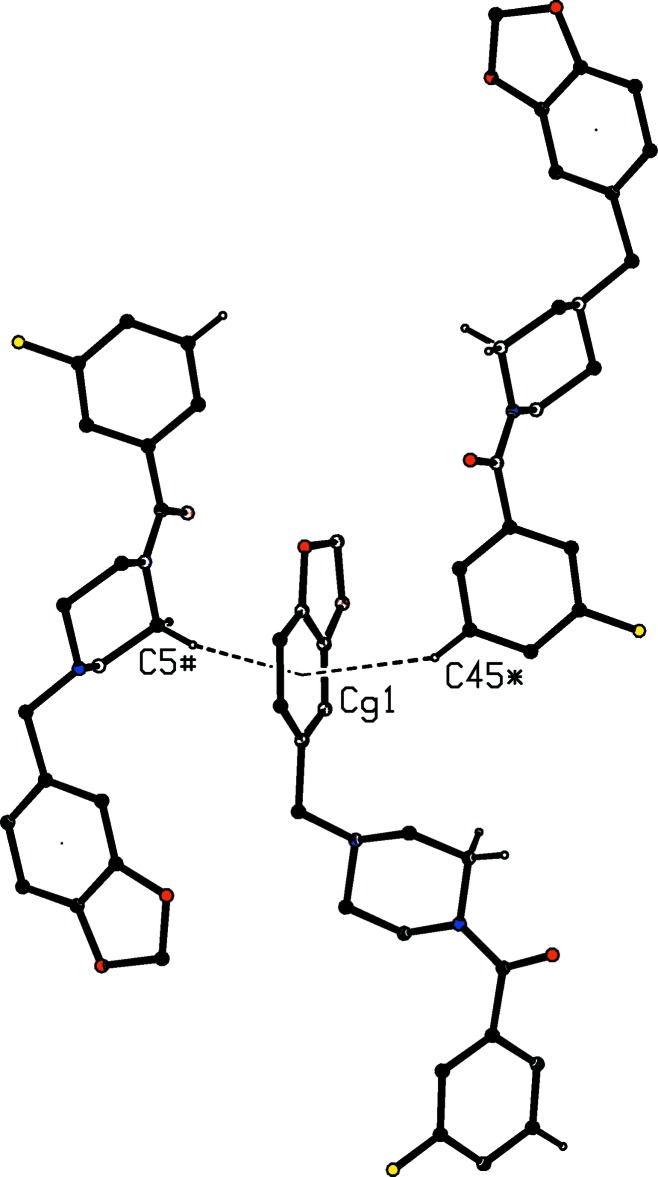
Part of the crystal structure of compound (I) showing the two C—H⋯π(arene) hydrogen bonds with a common aryl acceptor. The hydrogen bonds are drawn as dashed lines and, for the sake of clarity, the unit-cell outline and the H atoms bonded to the C atoms not involved in the motifs shown have been omitted. The atoms marked with an asterisk (*) or a hash (#) are at the symmetry positions (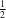 + *x*, 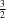 − *y*, −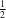 + *z*) and (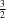 − *x*, −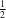 + *y*, 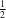 − *z*), respectively.

Despite their similar mol­ecular constitutions and conformations, compounds (I)–(III) all exhibit different types of direction-specific inter­molecular inter­actions. In the crystal structure of compound (I), a combination of one C—H⋯O hydrogen bond and two C—H⋯π(arene) hydrogen bonds (Table 1 ▸) links the mol­ecules into a three-dimensional framework structure, whose formation can readily be analysed in terms of simple sub-structures (Ferguson *et al.*, 1998*a* ▸,*b* ▸; Gregson *et al.*, 2000 ▸). The C—H⋯O hydrogen bond links mol­ecules related by the 2_1_ screw axis along (0.25, *y*, 0.25) to form a *C*(5) (Etter, 1990 ▸; Etter *et al.*, 1990 ▸; Bernstein *et al.*, 1995 ▸) chain running parallel to the [010] direction. In addition, the C—H⋯π(arene) hydrogen bond having atom C5 as the donor links mol­ecules related by the 2_1_ screw axis along (0.75, *y*, 0.25) into a second chain running parallel to [010] and, together, these two inter­actions generate a sheet lying parallel to (001) (Fig. 4 ▸). The second C—H⋯π(arene) hydrogen bond, having atom C45 as the donor, links mol­ecules related by the *n*-glide plane at *y* = 0.75 into a chain running parallel to the [10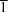] direction (Fig. 5 ▸), and chains of this type link the (001) sheets into a continuous three-dimensional structure. It is inter­esting to note that both C—H⋯π(arene) hydrogen bonds utilize the same ring as the acceptor, with one donor approaching each face of this ring (Fig. 6 ▸), with the angle H5^i^⋯*Cg*1⋯H45^ii^ = 152°, where *Cg*1 represents the centroid of the ring (C3*A*, C14, C15, C16, C17, C7*A*) and the symmetry codes are (i) 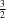 − *x*, −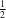 + *y*, 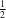 − *z*) and (ii) (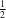 + *x*, 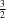 − *y*, −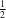 + *z*). Hence, the two mol­ecules providing the donor atoms here are related by inversion across (1, 1/2, 0). In this structure, the atoms of type O11 in the mol­ecules at (*x*, *y*, *z*) and (2 − *x*, 1 − *y*, −*z*) are separated by a distance of only 2.7888 (18) Å. At the same time, the atoms C12 and H12 at (*x*, *y*, *z*) are distant from O11 at (2 − *x*, 1 − *y*, −*z*) by 2.66 and 3.008 (2) Å, respectively, with an associated C—H⋯O angle of 101°; the H⋯O distance is too long and the C—H⋯O angle is too small for this contact to be regarded as a hydrogen bond, but the short O⋯O distance here is perhaps associated with this ‘failed’ hydrogen bond involving atom C12.

In contrast to the three-dimensional supra­molecular assembly in (I) generated by three hydrogen bonds, the only direction-specific inter­molecular inter­action in (II) is a single C—H⋯O contact, in which the *D–*-H⋯*A* angle is only 123° so that this cannot be regarded as structurally significant (Wood *et al.*, 2009 ▸). The only direction-specific inter­molecular inter­actions in (III) are a C—Cl⋯(ring) contact involving the 1,3-dioxolane ring, but since this ring is not aromatic, this contact cannot be regarded as structurally significant; and a short Cl⋯Cl contact between inversion-related pairs of mol­ecules. For the atoms of type Cl44 in the mol­ecules at (*x*, *y*, *z*) and (−*x*, −*y*, 2 − *z*), the Cl⋯Cl^i^ distance is 3.3963 (7) Å with an associated C—Cl⋯Cl^i^ angle of 137.68 (5)° [symmetry code: (i) −*x*, −*y*, 2 − *z*]. For C—Cl⋯Cl angles of 90 and 180°, values of 1.78 and 1.58 Å have been suggested (Nyburg & Faerman, 1985 ▸) for the major and minor van der Waals radii: on this basis, a value of around 1.68 Å would seem appropriate to a C—Cl⋯Cl angle close to 135°, so that the observed Cl⋯Cl contact distance in (III) is not exceptional, and is probably therefore of no structural significance. Thus for both (II) and (III), the mol­ecular packing depends solely on mol­ecular shape and van der Waals forces.

## Database survey   

It is of inter­est briefly to compare the supra­molecular assembly found here for compounds (I)–(III) with that observed in some related compounds. In 1-[(1,3-benzodioxol-5-yl)meth­yl]-4-(pyrimidin-2-yl)piperazine (piribedil), the mol­ecules are linked into sheets by three independent C—H⋯π hydrogen bonds (Wu *et al.*, 2013 ▸), and in 1-(2-iodo­benzo­yl)-4-(pyrimidin-2-yl)piperazine, the mol­ecules are linked by a combination of C—H⋯O and C—H⋯π hydrogen bonds to form a three-dimensional structure which is augmented by π–π stacking inter­actions and N⋯I inter­actions (Mahesha *et al.*, 2019 ▸). The amidic compound *N*-(4-chloro­phen­yl)-4-(pyrimidin-2-yl)piperazine-1-carboxamide crystallizes with *Z*′ = 2 in space group *P*2_1_/*c*, and the mol­ecules are linked by two independent N—H⋯O hydrogen bonds to form chains of 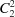
(8) type, although these are described as *C*(4) in the original report (Li, 2011 ▸). Finally, we note the structures of three salts derived by monoprotonation of the starting material 1-[(1,3-benzodioxol-5-yl)meth­yl]piperazine used in the synthesis of compounds (I)–(III): protonation occurs at the unsubstituted N atom of the piperazine unit in each of the picrate (Kavitha *et al.*, 2014*a* ▸), 4-nitro­benzoate (Kavitha *et al.*, 2014*b* ▸) and 4-chloro­benzoate (Kavitha *et al.*, 2014*c* ▸) salts, although the schematic diagrams given for the two carboxyl­ate salts depict protonation at the substituted N atom.

## Synthesis and crystallization   

1-[(1,3-Benzodioxol-5-yl)methyl]piperazine was purchased from Sigma–Aldrich and used as received. For the synthesis of compounds (I)–(III), 1-(3-di­methyl­amino­prop­yl)-3-ethyl­carbodimide (207 mg, 1.08 mmol), 1-hy­droxy­benzotriazole (121.6 mg, 0.9 mmol) and tri­ethyl­amine (0.5 ml, 3.7 mmol) were added to solutions of the appropriately substituted benzoic acid [3-fluoro­benzoic acid for (I), 2,6-di­fluoro­benzoic acid for (II) or 2,4-di­chloro­benzoic acid for (III)] (0.9 mmol) in *N*,*N*-di­methyl­formamide (5 ml) and the resulting mixtures were then stirred at 273 K for 20 min. A solution of 1-[(1,3-benzodioxol-5-yl)methyl]­piperazine (200 mg, 0.9 mmol) in *N*,*N*-di­methyl­formamide (5 ml) was then added to each mixture and stirring was continued overnight at ambient temperature. When the reactions were complete as confirmed using thin-layer chromatography, an excess of water was added to each of the mixtures, which were then exhaustively extracted using ethyl acetate. Each of the organic fractions was then washed successively with aqueous hydro­chloric acid (1 mol dm^−3^), then with a saturated aqueous solution of sodium hydrogencarbonate, and finally with brine. The organic fractions were then dried over anhydrous sodium sulfate and concentrated under reduced pressure. Slow evaporation of these solutions, at ambient temperature and in the presence of air, gave crystals of compounds (I)–(III) suitable for single-crystal X-ray diffraction: m.p. (I) 383–386 K, (II) 373 K, (III) 394–396 K.

## Refinement   

Crystal data, data collection and structure refinement details are summarized in Table 2 ▸. All H atoms were located in difference maps, and they were subsequently treated as riding atoms in geometrically idealized positions with C—H distances 0.95 Å (aromatic) or 0.99 Å (CH_2_) and with *U*_iso_(H) = 1.2*U*_eq_(C). For compound (I), fifteen bad outlier reflections were omitted from the data set. For compound (II), the correct orientation of the structure with respect to the polar axis direction could not be established because of the lack of significant resonant scattering: thus calculation of the Flack *x* parameter (Flack, 1983 ▸) using using 1369 quotients of the type [(*I* ^+^) − (*I* ^−^)]/[(*I* ^+^) + (*I* ^−^)] (Parsons *et al.*, 2013 ▸) gave a value −0.3 (10), which must be regarded as indeterminate (Flack & Bernardinelli, 2000 ▸), despite the 93% coverage of Friedel pairs, while the value of the Hooft *y* parameter (Hooft *et al.*, 2008 ▸), *y* = −0.2 (6), is likewise indeterminate.

## Supplementary Material

Crystal structure: contains datablock(s) global, I, II, III. DOI: 10.1107/S2056989019000458/zl2747sup1.cif

Structure factors: contains datablock(s) I. DOI: 10.1107/S2056989019000458/zl2747Isup2.hkl

Structure factors: contains datablock(s) II. DOI: 10.1107/S2056989019000458/zl2747IIsup3.hkl

Structure factors: contains datablock(s) III. DOI: 10.1107/S2056989019000458/zl2747IIIsup4.hkl

Supporting information file. DOI: 10.1107/S2056989019000458/zl2747Isup5.cml

Supporting information file. DOI: 10.1107/S2056989019000458/zl2747IIsup6.cml

Supporting information file. DOI: 10.1107/S2056989019000458/zl2747IIIsup7.cml

CCDC references: 1889708, 1889709, 1889710

Additional supporting information:  crystallographic information; 3D view; checkCIF report

## Figures and Tables

**Figure 2:**
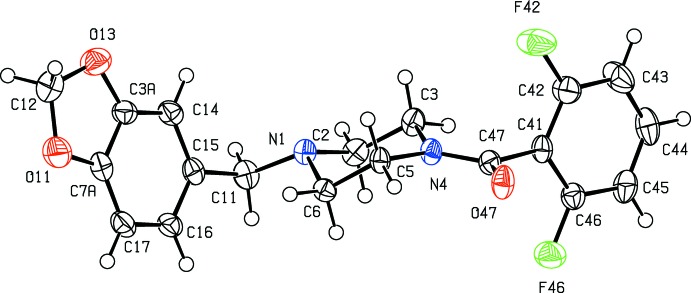
The mol­ecular structure of compound (II) showing the atom-labelling scheme. Displacement ellipsoids are drawn at the 50% probability level.

**Figure 3:**
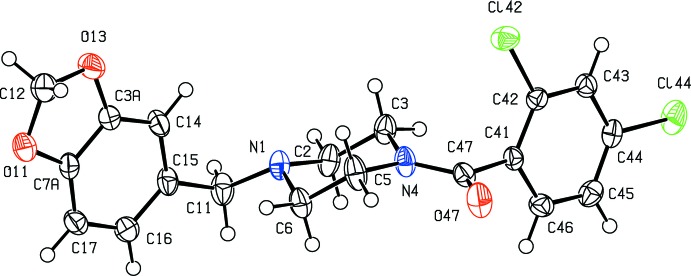
The mol­ecular structure of compound (III) showing the atom-labelling scheme. Displacement ellipsoids are drawn at the 50% probability level.

**Table 1:** Hydrogen-bond geometry (Å, °) for (I) *Cg*1 represents the centroid of the C3*A*, C14, C15, C16, C17, C7*A* ring.

*D*—H⋯*A*	*D*—H	H⋯*A*	*D*⋯*A*	*D*—H⋯*A*
C42—H42⋯O47^i^	0.95	2.34	3.273 (2)	168
C5—H5*A*⋯*Cg*1^ii^	0.99	2.76	3.7310 (18)	168
C45—H45⋯*Cg*1^iii^	0.95	2.90	3.7470 (18)	149

Symmetry codes: (i) 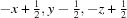; (ii) 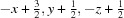; (iii) 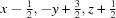.

**Table 2:** Experimental details

	(I)	(II)	(III)
Crystal data			
Chemical formula	C_19_H_19_FN_2_O_3_	C_19_H_18_F_2_N_2_O_3_	C_19_H_18_Cl_2_N_2_O_3_
*M*_r_	342.36	360.35	393.25
Crystal system, space group	Monoclinic, *P*2_1_/*n*	Orthorhombic, *P* *c* *a*2_1_	Monoclinic, *P*2_1_/*n*
Temperature (K)	173	173	173
*a*, *b*, *c* (Å)	12.2358 (16), 10.3185 (14), 14.2310 (19)	14.2762 (9), 15.9821 (10), 7.3753 (5)	12.2889 (14), 12.3034 (14), 13.3667 (15)
α, β, γ (°)	90, 111.199 (2), 90	90, 90, 90	90, 116.295 (1), 90
*V* (Å^3^)	1675.2 (4)	1682.78 (19)	1811.9 (4)
*Z*	4	4	4
Radiation type	Mo *K*α	Mo *K*α	Mo *K*α
μ (mm^−1^)	0.10	0.11	0.38
Crystal size (mm)	0.48 × 0.29 × 0.28	0.91 × 0.35 × 0.17	0.49 × 0.48 × 0.38

Data collection			
Diffractometer	Bruker APEXII CCD	Bruker APEXII CCD	Bruker APEXII CCD
Absorption correction	Multi-scan (*SADABS*; Bruker, 2015 ▸)	Multi-scan (*SADABS*; Bruker, 2015 ▸)	Multi-scan (*SADABS*; Bruker, 2015 ▸)
*T*_min_, *T*_max_	0.813, 0.972	0.587, 0.981	0.776, 0.867
No. of measured, independent and observed [*I* > 2σ(*I*)] reflections	8635, 3674, 2975	9016, 3743, 3449	9718, 4054, 3545
*R*_int_	0.021	0.057	0.017
(sin θ/λ)_max_ (Å^−1^)	0.651	0.650	0.648

Refinement			
*R*[*F* ^2^ > 2σ(*F* ^2^)], *wR*(*F* ^2^), *S*	0.040, 0.114, 1.10	0.054, 0.155, 1.16	0.031, 0.087, 1.04
No. of reflections	3674	3743	4054
No. of parameters	226	235	235
No. of restraints	0	1	0
H-atom treatment	H-atom parameters constrained	H-atom parameters constrained	H-atom parameters constrained
Δρ_max_, Δρ_min_ (e Å^−3^)	0.24, −0.18	0.17, −0.22	0.37, −0.38
Absolute structure	–	Flack *x* determined using 1369 quotients [(*I* ^+^)−(*I* ^−^)]/[(*I* ^+^)+(*I* ^−^)] (Parsons *et al.*, 2013 ▸)	–

Computer programs: *APEX2* (Bruker, 2004 ▸) *SAINT* (Bruker, 2013 ▸), *SHELXS97* (Sheldrick, 2015 ▸), *SHELXL2014* (Sheldrick,2015 ▸) and *PLATON* (Spek, 2009 ▸).

## supplementary crystallographic information

### 1-[(1,3-Benzodioxol-5-yl)methyl]-4-(3-fluorobenzoyl)piperazine (I) Crystal data

**Table:**

C_19_H_19_FN_2_O_3_	*F*(000) = 720
*M**_r_* = 342.36	*D*_x_ = 1.357 Mg m^−^^3^
Monoclinic, *P*2_1_/*n*	Mo *K*α radiation, λ = 0.71073 Å
*a* = 12.2358 (16) Å	Cell parameters from 3689 reflections
*b* = 10.3185 (14) Å	θ = 1.9–27.6°
*c* = 14.2310 (19) Å	µ = 0.10 mm^−^^1^
β = 111.199 (2)°	*T* = 173 K
*V* = 1675.2 (4) Å^3^	Block, colourless
*Z* = 4	0.48 × 0.29 × 0.28 mm

### 1-[(1,3-Benzodioxol-5-yl)methyl]-4-(3-fluorobenzoyl)piperazine (I) Data collection

**Table:**

Bruker APEXII CCD diffractometer	3674 independent reflections
Radiation source: fine focus sealed tube	2975 reflections with *I* > 2σ(*I*)
Graphite monochromator	*R*_int_ = 0.021
Detector resolution: 0.3333 pixels mm^-1^	θ_max_ = 27.6°, θ_min_ = 1.9°
φ and ω scans	*h* = −10→15
Absorption correction: multi-scan (SADABS; Bruker, 2015)	*k* = −13→13
*T*_min_ = 0.813, *T*_max_ = 0.972	*l* = −18→9
8635 measured reflections	

### 1-[(1,3-Benzodioxol-5-yl)methyl]-4-(3-fluorobenzoyl)piperazine (I) Refinement

**Table:**

Refinement on *F*^2^	0 restraints
Least-squares matrix: full	Hydrogen site location: inferred from neighbouring sites
*R*[*F*^2^ > 2σ(*F*^2^)] = 0.040	H-atom parameters constrained
*wR*(*F*^2^) = 0.114	*w* = 1/[σ^2^(*F*_o_^2^) + (0.0476*P*)^2^ + 0.4713*P*] where *P* = (*F*_o_^2^ + 2*F*_c_^2^)/3
*S* = 1.10	(Δ/σ)_max_ < 0.001
3674 reflections	Δρ_max_ = 0.24 e Å^−^^3^
226 parameters	Δρ_min_ = −0.18 e Å^−^^3^

### 1-[(1,3-Benzodioxol-5-yl)methyl]-4-(3-fluorobenzoyl)piperazine (I) Special details

**Table:**

Geometry. All esds (except the esd in the dihedral angle between two l.s. planes) are estimated using the full covariance matrix. The cell esds are taken into account individually in the estimation of esds in distances, angles and torsion angles; correlations between esds in cell parameters are only used when they are defined by crystal symmetry. An approximate (isotropic) treatment of cell esds is used for estimating esds involving l.s. planes.

### 1-[(1,3-Benzodioxol-5-yl)methyl]-4-(3-fluorobenzoyl)piperazine (I) Fractional atomic coordinates and isotropic or equivalent isotropic displacement parameters (Å^2^)

**Table:**

	*x*	*y*	*z*	*U*_iso_*/*U*_eq_	
N1	0.57212 (10)	0.47476 (11)	0.26954 (9)	0.0303 (3)	
C2	0.51223 (13)	0.44782 (14)	0.33984 (11)	0.0330 (3)	
H2A	0.5622	0.3918	0.3952	0.040*	
H2B	0.4379	0.4014	0.3043	0.040*	
C3	0.48694 (13)	0.57400 (15)	0.38272 (11)	0.0356 (3)	
H3A	0.4438	0.5560	0.4283	0.043*	
H3B	0.5616	0.6173	0.4224	0.043*	
N4	0.41697 (11)	0.65903 (12)	0.30093 (9)	0.0327 (3)	
C5	0.47088 (13)	0.68273 (14)	0.22552 (11)	0.0327 (3)	
H5A	0.5444	0.7325	0.2564	0.039*	
H5B	0.4170	0.7344	0.1691	0.039*	
C6	0.49704 (13)	0.55452 (14)	0.18609 (11)	0.0313 (3)	
H6A	0.4228	0.5081	0.1502	0.038*	
H6B	0.5368	0.5704	0.1377	0.038*	
C11	0.60711 (14)	0.35498 (14)	0.23287 (12)	0.0368 (3)	
H11A	0.5367	0.3124	0.1845	0.044*	
H11B	0.6424	0.2951	0.2903	0.044*	
O11	0.95161 (10)	0.44449 (12)	0.06543 (9)	0.0460 (3)	
C12	1.01387 (15)	0.54464 (18)	0.13322 (13)	0.0450 (4)	
H12A	1.0984	0.5232	0.1624	0.054*	
H12B	1.0049	0.6279	0.0967	0.054*	
O13	0.96722 (10)	0.55552 (12)	0.21141 (9)	0.0453 (3)	
C3A	0.86787 (13)	0.48056 (14)	0.18111 (11)	0.0317 (3)	
C14	0.78769 (13)	0.46591 (14)	0.22718 (11)	0.0323 (3)	
H14	0.7950	0.5118	0.2870	0.039*	
C15	0.69410 (13)	0.37999 (14)	0.18201 (11)	0.0327 (3)	
C16	0.68486 (14)	0.31574 (15)	0.09369 (12)	0.0384 (4)	
H16	0.6203	0.2592	0.0636	0.046*	
C17	0.76762 (14)	0.33156 (16)	0.04750 (12)	0.0399 (4)	
H17	0.7610	0.2869	−0.0127	0.048*	
C7A	0.85801 (14)	0.41432 (14)	0.09347 (11)	0.0347 (3)	
C47	0.32103 (13)	0.72461 (14)	0.29797 (11)	0.0328 (3)	
O47	0.27475 (12)	0.80626 (13)	0.23290 (10)	0.0583 (4)	
C41	0.26784 (12)	0.69823 (13)	0.37650 (11)	0.0291 (3)	
C42	0.22151 (13)	0.57722 (14)	0.38544 (11)	0.0333 (3)	
H42	0.2306	0.5049	0.3476	0.040*	
C43	0.16209 (14)	0.56607 (15)	0.45098 (12)	0.0367 (3)	
F43	0.11374 (10)	0.44972 (10)	0.45775 (9)	0.0590 (3)	
C44	0.14867 (13)	0.66616 (16)	0.50952 (12)	0.0377 (4)	
H44	0.1079	0.6542	0.5544	0.045*	
C45	0.19660 (13)	0.78531 (16)	0.50088 (12)	0.0381 (4)	
H45	0.1896	0.8563	0.5409	0.046*	
C46	0.25459 (13)	0.80141 (14)	0.43428 (12)	0.0345 (3)	
H46	0.2857	0.8839	0.4280	0.041*	

### 1-[(1,3-Benzodioxol-5-yl)methyl]-4-(3-fluorobenzoyl)piperazine (I) Atomic displacement parameters (Å^2^)

**Table:**

	*U*^11^	*U*^22^	*U*^33^	*U*^12^	*U*^13^	*U*^23^
N1	0.0328 (6)	0.0277 (6)	0.0323 (6)	0.0018 (5)	0.0140 (5)	0.0033 (5)
C2	0.0307 (7)	0.0341 (8)	0.0338 (8)	0.0021 (6)	0.0113 (6)	0.0106 (6)
C3	0.0349 (8)	0.0444 (9)	0.0275 (7)	0.0076 (6)	0.0114 (6)	0.0080 (6)
N4	0.0373 (7)	0.0345 (7)	0.0283 (6)	0.0068 (5)	0.0145 (5)	0.0068 (5)
C5	0.0388 (8)	0.0300 (7)	0.0317 (7)	0.0015 (6)	0.0157 (6)	0.0061 (6)
C6	0.0350 (8)	0.0311 (7)	0.0285 (7)	−0.0005 (6)	0.0121 (6)	0.0025 (6)
C11	0.0408 (8)	0.0276 (7)	0.0427 (9)	0.0007 (6)	0.0160 (7)	0.0009 (6)
O11	0.0512 (7)	0.0504 (7)	0.0447 (7)	−0.0015 (5)	0.0275 (6)	−0.0093 (5)
C12	0.0400 (9)	0.0524 (10)	0.0469 (10)	0.0013 (7)	0.0209 (8)	−0.0054 (8)
O13	0.0426 (6)	0.0511 (7)	0.0478 (7)	−0.0086 (5)	0.0231 (6)	−0.0146 (5)
C3A	0.0325 (7)	0.0279 (7)	0.0318 (7)	0.0059 (6)	0.0082 (6)	−0.0006 (6)
C14	0.0367 (8)	0.0306 (7)	0.0290 (7)	0.0048 (6)	0.0113 (6)	−0.0022 (6)
C15	0.0351 (8)	0.0273 (7)	0.0344 (8)	0.0064 (6)	0.0108 (6)	0.0013 (6)
C16	0.0358 (8)	0.0344 (8)	0.0391 (8)	0.0030 (6)	0.0066 (7)	−0.0068 (6)
C17	0.0465 (9)	0.0388 (8)	0.0324 (8)	0.0077 (7)	0.0117 (7)	−0.0073 (6)
C7A	0.0401 (8)	0.0330 (8)	0.0330 (8)	0.0109 (6)	0.0160 (7)	0.0023 (6)
C47	0.0369 (8)	0.0260 (7)	0.0370 (8)	0.0018 (6)	0.0150 (6)	0.0059 (6)
O47	0.0624 (8)	0.0576 (8)	0.0686 (9)	0.0303 (6)	0.0401 (7)	0.0385 (7)
C41	0.0263 (7)	0.0280 (7)	0.0303 (7)	0.0019 (5)	0.0071 (6)	0.0036 (5)
C42	0.0400 (8)	0.0268 (7)	0.0329 (7)	−0.0001 (6)	0.0128 (7)	0.0002 (6)
C43	0.0383 (8)	0.0311 (8)	0.0398 (8)	−0.0047 (6)	0.0130 (7)	0.0068 (6)
F43	0.0759 (8)	0.0378 (6)	0.0757 (7)	−0.0142 (5)	0.0424 (6)	0.0061 (5)
C44	0.0342 (8)	0.0466 (9)	0.0337 (8)	0.0027 (7)	0.0141 (7)	0.0058 (7)
C45	0.0368 (8)	0.0382 (8)	0.0387 (8)	0.0020 (6)	0.0127 (7)	−0.0064 (7)
C46	0.0332 (8)	0.0270 (7)	0.0431 (8)	−0.0020 (6)	0.0135 (7)	−0.0006 (6)

### 1-[(1,3-Benzodioxol-5-yl)methyl]-4-(3-fluorobenzoyl)piperazine (I) Geometric parameters (Å, º)

**Table:**

N1—C6	1.4638 (18)	O13—C3A	1.3722 (18)
N1—C11	1.4645 (18)	C3A—C14	1.371 (2)
N1—C2	1.4647 (18)	C3A—C7A	1.389 (2)
C2—C3	1.517 (2)	C14—C15	1.406 (2)
C2—H2A	0.9900	C14—H14	0.9500
C2—H2B	0.9900	C15—C16	1.389 (2)
C3—N4	1.4619 (18)	C16—C17	1.402 (2)
C3—H3A	0.9900	C16—H16	0.9500
C3—H3B	0.9900	C17—C7A	1.363 (2)
N4—C47	1.3423 (19)	C17—H17	0.9500
N4—C5	1.4692 (17)	C47—O47	1.2283 (18)
C5—C6	1.5156 (19)	C47—C41	1.507 (2)
C5—H5A	0.9900	C41—C46	1.390 (2)
C5—H5B	0.9900	C41—C42	1.3960 (19)
C6—H6A	0.9900	C42—C43	1.379 (2)
C6—H6B	0.9900	C42—H42	0.9500
C11—C15	1.510 (2)	C43—F43	1.3572 (17)
C11—H11A	0.9900	C43—C44	1.374 (2)
C11—H11B	0.9900	C44—C45	1.387 (2)
O11—C7A	1.3777 (19)	C44—H44	0.9500
O11—C12	1.431 (2)	C45—C46	1.384 (2)
C12—O13	1.4268 (19)	C45—H45	0.9500
C12—H12A	0.9900	C46—H46	0.9500
C12—H12B	0.9900		
			
C6—N1—C11	111.35 (11)	H12A—C12—H12B	108.4
C6—N1—C2	109.80 (11)	C3A—O13—C12	105.63 (12)
C11—N1—C2	111.48 (11)	C14—C3A—O13	128.14 (13)
N1—C2—C3	109.70 (12)	C14—C3A—C7A	122.03 (14)
N1—C2—H2A	109.7	O13—C3A—C7A	109.82 (13)
C3—C2—H2A	109.7	C3A—C14—C15	117.14 (13)
N1—C2—H2B	109.7	C3A—C14—H14	121.4
C3—C2—H2B	109.7	C15—C14—H14	121.4
H2A—C2—H2B	108.2	C16—C15—C14	120.05 (14)
N4—C3—C2	109.93 (12)	C16—C15—C11	120.75 (14)
N4—C3—H3A	109.7	C14—C15—C11	119.16 (13)
C2—C3—H3A	109.7	C15—C16—C17	122.17 (15)
N4—C3—H3B	109.7	C15—C16—H16	118.9
C2—C3—H3B	109.7	C17—C16—H16	118.9
H3A—C3—H3B	108.2	C7A—C17—C16	116.51 (14)
C47—N4—C3	125.54 (12)	C7A—C17—H17	121.7
C47—N4—C5	120.78 (12)	C16—C17—H17	121.7
C3—N4—C5	113.15 (11)	C17—C7A—O11	128.17 (14)
N4—C5—C6	109.62 (11)	C17—C7A—C3A	122.10 (14)
N4—C5—H5A	109.7	O11—C7A—C3A	109.73 (14)
C6—C5—H5A	109.7	O47—C47—N4	121.94 (13)
N4—C5—H5B	109.7	O47—C47—C41	118.54 (13)
C6—C5—H5B	109.7	N4—C47—C41	119.52 (12)
H5A—C5—H5B	108.2	C46—C41—C42	119.54 (13)
N1—C6—C5	110.19 (12)	C46—C41—C47	118.31 (12)
N1—C6—H6A	109.6	C42—C41—C47	121.84 (13)
C5—C6—H6A	109.6	C43—C42—C41	117.84 (13)
N1—C6—H6B	109.6	C43—C42—H42	121.1
C5—C6—H6B	109.6	C41—C42—H42	121.1
H6A—C6—H6B	108.1	F43—C43—C44	118.11 (14)
N1—C11—C15	111.97 (12)	F43—C43—C42	118.15 (14)
N1—C11—H11A	109.2	C44—C43—C42	123.73 (14)
C15—C11—H11A	109.2	C43—C44—C45	117.76 (14)
N1—C11—H11B	109.2	C43—C44—H44	121.1
C15—C11—H11B	109.2	C45—C44—H44	121.1
H11A—C11—H11B	107.9	C46—C45—C44	120.34 (14)
C7A—O11—C12	105.29 (11)	C46—C45—H45	119.8
O13—C12—O11	108.48 (13)	C44—C45—H45	119.8
O13—C12—H12A	110.0	C45—C46—C41	120.77 (14)
O11—C12—H12A	110.0	C45—C46—H46	119.6
O13—C12—H12B	110.0	C41—C46—H46	119.6
O11—C12—H12B	110.0		
			
C6—N1—C2—C3	−60.96 (15)	C16—C17—C7A—C3A	−0.4 (2)
C11—N1—C2—C3	175.17 (12)	C12—O11—C7A—C17	174.64 (16)
N1—C2—C3—N4	57.23 (15)	C12—O11—C7A—C3A	−6.53 (16)
C2—C3—N4—C47	133.38 (15)	C14—C3A—C7A—C17	0.5 (2)
C2—C3—N4—C5	−55.00 (16)	O13—C3A—C7A—C17	179.30 (14)
C47—N4—C5—C6	−133.39 (14)	C14—C3A—C7A—O11	−178.38 (13)
C3—N4—C5—C6	54.54 (16)	O13—C3A—C7A—O11	0.39 (17)
C11—N1—C6—C5	−175.10 (11)	C3—N4—C47—O47	171.03 (16)
C2—N1—C6—C5	60.95 (15)	C5—N4—C47—O47	0.0 (2)
N4—C5—C6—N1	−56.63 (15)	C3—N4—C47—C41	−8.9 (2)
C6—N1—C11—C15	71.31 (15)	C5—N4—C47—C41	−179.92 (13)
C2—N1—C11—C15	−165.70 (12)	O47—C47—C41—C46	−56.5 (2)
C7A—O11—C12—O13	10.19 (17)	N4—C47—C41—C46	123.39 (16)
O11—C12—O13—C3A	−9.99 (17)	O47—C47—C41—C42	116.97 (17)
C12—O13—C3A—C14	−175.37 (15)	N4—C47—C41—C42	−63.11 (19)
C12—O13—C3A—C7A	5.95 (17)	C46—C41—C42—C43	1.1 (2)
O13—C3A—C14—C15	−178.36 (14)	C47—C41—C42—C43	−172.33 (13)
C7A—C3A—C14—C15	0.2 (2)	C41—C42—C43—F43	178.02 (14)
C3A—C14—C15—C16	−0.9 (2)	C41—C42—C43—C44	−1.7 (2)
C3A—C14—C15—C11	176.73 (13)	F43—C43—C44—C45	−178.92 (14)
N1—C11—C15—C16	−138.33 (14)	C42—C43—C44—C45	0.8 (2)
N1—C11—C15—C14	44.02 (18)	C43—C44—C45—C46	0.7 (2)
C14—C15—C16—C17	1.1 (2)	C44—C45—C46—C41	−1.3 (2)
C11—C15—C16—C17	−176.55 (14)	C42—C41—C46—C45	0.4 (2)
C15—C16—C17—C7A	−0.4 (2)	C47—C41—C46—C45	174.01 (13)
C16—C17—C7A—O11	178.28 (14)		

### 1-[(1,3-Benzodioxol-5-yl)methyl]-4-(3-fluorobenzoyl)piperazine (I) Hydrogen-bond geometry (Å, º)

*Cg*1 represents the centroid of the C3*A*, C14, C15, C16, C17, C7*A* ring.

**Table:**

*D*—H···*A*	*D*—H	H···*A*	*D*···*A*	*D*—H···*A*
C42—H42···O47^i^	0.95	2.34	3.273 (2)	168
C5—H5*A*···*Cg*1^ii^	0.99	2.76	3.7310 (18)	168
C45—H45···*Cg*1^iii^	0.95	2.90	3.7470 (18)	149

Symmetry codes: (i) −*x*+1/2, *y*−1/2, −*z*+1/2; (ii) −*x*+3/2, *y*+1/2, −*z*+1/2; (iii) *x*−1/2, −*y*+3/2, *z*+1/2.

### 1-[(1,3-Benzodioxol-5-yl)methyl]-4-(2,6-difluorobenzoyl)piperazine (II) Crystal data

**Table:**

C_19_H_18_F_2_N_2_O_3_	*D*_x_ = 1.422 Mg m^−^^3^
*M**_r_* = 360.35	Mo *K*α radiation, λ = 0.71073 Å
Orthorhombic, *P**c**a*2_1_	Cell parameters from 3743 reflections
*a* = 14.2762 (9) Å	θ = 1.9–27.5°
*b* = 15.9821 (10) Å	µ = 0.11 mm^−^^1^
*c* = 7.3753 (5) Å	*T* = 173 K
*V* = 1682.78 (19) Å^3^	Needle, colourless
*Z* = 4	0.91 × 0.35 × 0.17 mm
*F*(000) = 752	

### 1-[(1,3-Benzodioxol-5-yl)methyl]-4-(2,6-difluorobenzoyl)piperazine (II) Data collection

**Table:**

Bruker APEXII CCD diffractometer	3743 independent reflections
Radiation source: fine focus sealed tube	3449 reflections with *I* > 2σ(*I*)
Graphite monochromator	*R*_int_ = 0.057
Detector resolution: 0.3333 pixels mm^-1^	θ_max_ = 27.5°, θ_min_ = 1.9°
φ and ω scans	*h* = −14→18
Absorption correction: multi-scan (SADABS; Bruker, 2015)	*k* = −16→20
*T*_min_ = 0.587, *T*_max_ = 0.981	*l* = −9→9
9016 measured reflections	

### 1-[(1,3-Benzodioxol-5-yl)methyl]-4-(2,6-difluorobenzoyl)piperazine (II) Refinement

**Table:**

Refinement on *F*^2^	Hydrogen site location: inferred from neighbouring sites
Least-squares matrix: full	H-atom parameters constrained
*R*[*F*^2^ > 2σ(*F*^2^)] = 0.054	*w* = 1/[σ^2^(*F*_o_^2^) + (0.096*P*)^2^] where *P* = (*F*_o_^2^ + 2*F*_c_^2^)/3
*wR*(*F*^2^) = 0.155	(Δ/σ)_max_ < 0.001
*S* = 1.16	Δρ_max_ = 0.17 e Å^−^^3^
3743 reflections	Δρ_min_ = −0.21 e Å^−^^3^
235 parameters	Absolute structure: Flack *x* determined using 1369 quotients [(*I*^+^)-(*I*^-^)]/[(*I*^+^)+(*I*^-^)] (Parsons *et al.*, 2013)
1 restraint	

### 1-[(1,3-Benzodioxol-5-yl)methyl]-4-(2,6-difluorobenzoyl)piperazine (II) Special details

**Table:**

Geometry. All esds (except the esd in the dihedral angle between two l.s. planes) are estimated using the full covariance matrix. The cell esds are taken into account individually in the estimation of esds in distances, angles and torsion angles; correlations between esds in cell parameters are only used when they are defined by crystal symmetry. An approximate (isotropic) treatment of cell esds is used for estimating esds involving l.s. planes.

### 1-[(1,3-Benzodioxol-5-yl)methyl]-4-(2,6-difluorobenzoyl)piperazine (II) Fractional atomic coordinates and isotropic or equivalent isotropic displacement parameters (Å^2^)

**Table:**

	*x*	*y*	*z*	*U*_iso_*/*U*_eq_	
N1	0.36051 (17)	0.62727 (15)	0.3017 (3)	0.0249 (5)	
C2	0.3856 (2)	0.71107 (18)	0.2369 (5)	0.0302 (6)	
H2A	0.3597	0.7197	0.1138	0.036*	
H2B	0.4546	0.7163	0.2299	0.036*	
C3	0.3470 (2)	0.77682 (17)	0.3641 (4)	0.0302 (6)	
H3A	0.3686	0.8328	0.3249	0.036*	
H3B	0.2777	0.7762	0.3590	0.036*	
N4	0.37766 (17)	0.76146 (14)	0.5509 (4)	0.0265 (5)	
C5	0.3658 (2)	0.67513 (15)	0.6138 (4)	0.0247 (5)	
H5A	0.2984	0.6633	0.6317	0.030*	
H5B	0.3979	0.6679	0.7317	0.030*	
C6	0.40582 (18)	0.61456 (18)	0.4776 (4)	0.0243 (6)	
H6A	0.4742	0.6236	0.4659	0.029*	
H6B	0.3953	0.5564	0.5193	0.029*	
C11	0.3880 (2)	0.5620 (2)	0.1720 (4)	0.0320 (7)	
H11A	0.4569	0.5628	0.1566	0.038*	
H11B	0.3592	0.5740	0.0526	0.038*	
O11	0.26117 (18)	0.25056 (13)	0.4272 (4)	0.0445 (6)	
C12	0.1638 (3)	0.2617 (2)	0.3896 (6)	0.0426 (8)	
H12A	0.1431	0.2208	0.2971	0.051*	
H12B	0.1266	0.2526	0.5011	0.051*	
O13	0.15012 (16)	0.34454 (15)	0.3247 (4)	0.0436 (6)	
C3A	0.2382 (2)	0.37818 (18)	0.3049 (5)	0.0295 (6)	
C14	0.2621 (2)	0.45542 (17)	0.2400 (4)	0.0299 (6)	
H14	0.2157	0.4937	0.1993	0.036*	
C15	0.35761 (19)	0.47614 (18)	0.2356 (4)	0.0276 (6)	
C16	0.4231 (2)	0.4189 (2)	0.2983 (4)	0.0309 (6)	
H16	0.4875	0.4339	0.2961	0.037*	
C17	0.3983 (2)	0.34019 (19)	0.3646 (5)	0.0336 (7)	
H17	0.4438	0.3015	0.4067	0.040*	
C7A	0.3045 (2)	0.32183 (18)	0.3656 (4)	0.0317 (6)	
C47	0.4015 (2)	0.82057 (17)	0.6718 (4)	0.0262 (6)	
O47	0.41835 (17)	0.80559 (13)	0.8317 (3)	0.0366 (5)	
C41	0.4064 (2)	0.91022 (16)	0.6064 (5)	0.0277 (6)	
C42	0.3281 (2)	0.96071 (19)	0.5945 (6)	0.0384 (8)	
F42	0.24359 (14)	0.92331 (12)	0.6222 (4)	0.0620 (8)	
C43	0.3316 (3)	1.0447 (2)	0.5555 (7)	0.0476 (9)	
H43	0.2760	1.0772	0.5492	0.057*	
C44	0.4181 (3)	1.0804 (2)	0.5258 (5)	0.0432 (9)	
H44	0.4221	1.1383	0.4980	0.052*	
C45	0.4992 (2)	1.03346 (19)	0.5357 (5)	0.0375 (8)	
H45	0.5587	1.0584	0.5158	0.045*	
C46	0.4913 (2)	0.94955 (18)	0.5754 (5)	0.0303 (6)	
F46	0.56966 (12)	0.90206 (13)	0.5843 (4)	0.0464 (6)	

### 1-[(1,3-Benzodioxol-5-yl)methyl]-4-(2,6-difluorobenzoyl)piperazine (II) Atomic displacement parameters (Å^2^)

**Table:**

	*U*^11^	*U*^22^	*U*^33^	*U*^12^	*U*^13^	*U*^23^
N1	0.0308 (11)	0.0227 (11)	0.0214 (11)	−0.0015 (9)	−0.0005 (9)	0.0023 (9)
C2	0.0356 (14)	0.0296 (15)	0.0254 (14)	−0.0041 (12)	−0.0029 (12)	0.0090 (13)
C3	0.0399 (14)	0.0214 (12)	0.0293 (16)	−0.0025 (11)	−0.0088 (12)	0.0068 (12)
N4	0.0347 (11)	0.0173 (10)	0.0274 (13)	−0.0015 (9)	−0.0039 (10)	0.0052 (9)
C5	0.0328 (12)	0.0169 (11)	0.0243 (13)	−0.0024 (10)	−0.0002 (11)	0.0029 (11)
C6	0.0267 (12)	0.0220 (13)	0.0241 (14)	0.0012 (10)	0.0000 (11)	0.0044 (11)
C11	0.0400 (15)	0.0328 (15)	0.0232 (14)	0.0002 (13)	0.0051 (12)	−0.0003 (12)
O11	0.0469 (14)	0.0305 (11)	0.0563 (17)	0.0005 (10)	−0.0008 (12)	0.0081 (11)
C12	0.0465 (19)	0.0387 (17)	0.043 (2)	−0.0081 (14)	−0.0024 (15)	−0.0013 (15)
O13	0.0332 (11)	0.0427 (13)	0.0547 (16)	−0.0046 (10)	−0.0009 (11)	0.0050 (12)
C3A	0.0282 (13)	0.0343 (14)	0.0260 (14)	0.0040 (12)	−0.0012 (11)	−0.0043 (12)
C14	0.0338 (13)	0.0308 (13)	0.0252 (14)	0.0072 (12)	−0.0048 (12)	−0.0008 (13)
C15	0.0371 (14)	0.0266 (13)	0.0191 (12)	0.0031 (11)	0.0018 (11)	−0.0044 (11)
C16	0.0283 (12)	0.0336 (16)	0.0307 (15)	0.0059 (11)	0.0018 (12)	−0.0063 (13)
C17	0.0374 (14)	0.0270 (14)	0.0365 (17)	0.0093 (12)	−0.0025 (13)	−0.0011 (13)
C7A	0.0420 (15)	0.0242 (13)	0.0289 (16)	0.0036 (12)	0.0004 (13)	−0.0027 (12)
C47	0.0293 (11)	0.0188 (12)	0.0304 (15)	−0.0014 (10)	−0.0014 (11)	0.0043 (11)
O47	0.0560 (13)	0.0233 (10)	0.0305 (13)	−0.0055 (9)	−0.0064 (10)	0.0021 (9)
C41	0.0365 (14)	0.0183 (12)	0.0284 (15)	−0.0031 (11)	−0.0002 (12)	0.0023 (11)
C42	0.0344 (14)	0.0295 (16)	0.051 (2)	−0.0016 (12)	0.0038 (14)	0.0095 (15)
F42	0.0323 (9)	0.0451 (11)	0.109 (2)	−0.0006 (10)	0.0056 (12)	0.0293 (14)
C43	0.0502 (19)	0.0283 (16)	0.064 (3)	0.0091 (14)	0.0094 (18)	0.0128 (16)
C44	0.068 (2)	0.0187 (14)	0.043 (2)	−0.0038 (14)	0.0079 (17)	0.0061 (13)
C45	0.0461 (17)	0.0282 (14)	0.038 (2)	−0.0127 (14)	0.0074 (13)	0.0019 (14)
C46	0.0331 (14)	0.0265 (13)	0.0313 (17)	−0.0021 (11)	0.0026 (12)	0.0003 (13)
F46	0.0330 (9)	0.0404 (11)	0.0658 (16)	0.0006 (8)	0.0054 (10)	0.0060 (11)

### 1-[(1,3-Benzodioxol-5-yl)methyl]-4-(2,6-difluorobenzoyl)piperazine (II) Geometric parameters (Å, º)

**Table:**

N1—C6	1.464 (4)	O13—C3A	1.375 (4)
N1—C2	1.466 (3)	C3A—C14	1.367 (4)
N1—C11	1.469 (4)	C3A—C7A	1.381 (4)
C2—C3	1.513 (4)	C14—C15	1.404 (4)
C2—H2A	0.9900	C14—H14	0.9500
C2—H2B	0.9900	C15—C16	1.388 (4)
C3—N4	1.466 (4)	C16—C17	1.395 (5)
C3—H3A	0.9900	C16—H16	0.9500
C3—H3B	0.9900	C17—C7A	1.370 (4)
N4—C47	1.343 (4)	C17—H17	0.9500
N4—C5	1.465 (3)	C47—O47	1.227 (4)
C5—C6	1.507 (4)	C47—C41	1.513 (4)
C5—H5A	0.9900	C41—C42	1.382 (4)
C5—H5B	0.9900	C41—C46	1.384 (4)
C6—H6A	0.9900	C42—F42	1.362 (3)
C6—H6B	0.9900	C42—C43	1.373 (4)
C11—C15	1.514 (4)	C43—C44	1.379 (5)
C11—H11A	0.9900	C43—H43	0.9500
C11—H11B	0.9900	C44—C45	1.381 (5)
O11—C7A	1.374 (4)	C44—H44	0.9500
O11—C12	1.429 (4)	C45—C46	1.377 (4)
C12—O13	1.422 (4)	C45—H45	0.9500
C12—H12A	0.9900	C46—F46	1.354 (3)
C12—H12B	0.9900		
			
C6—N1—C2	107.9 (2)	H12A—C12—H12B	108.4
C6—N1—C11	111.2 (2)	C3A—O13—C12	105.9 (2)
C2—N1—C11	111.8 (2)	C14—C3A—O13	128.2 (3)
N1—C2—C3	110.1 (3)	C14—C3A—C7A	122.1 (3)
N1—C2—H2A	109.6	O13—C3A—C7A	109.7 (3)
C3—C2—H2A	109.6	C3A—C14—C15	117.6 (3)
N1—C2—H2B	109.6	C3A—C14—H14	121.2
C3—C2—H2B	109.6	C15—C14—H14	121.2
H2A—C2—H2B	108.2	C16—C15—C14	119.4 (3)
N4—C3—C2	111.0 (2)	C16—C15—C11	120.5 (3)
N4—C3—H3A	109.4	C14—C15—C11	120.0 (3)
C2—C3—H3A	109.4	C15—C16—C17	122.6 (3)
N4—C3—H3B	109.4	C15—C16—H16	118.7
C2—C3—H3B	109.4	C17—C16—H16	118.7
H3A—C3—H3B	108.0	C7A—C17—C16	116.3 (3)
C47—N4—C5	118.8 (3)	C7A—C17—H17	121.8
C47—N4—C3	125.6 (2)	C16—C17—H17	121.8
C5—N4—C3	114.9 (2)	C17—C7A—O11	128.3 (3)
N4—C5—C6	110.5 (2)	C17—C7A—C3A	121.9 (3)
N4—C5—H5A	109.6	O11—C7A—C3A	109.8 (3)
C6—C5—H5A	109.6	O47—C47—N4	123.4 (3)
N4—C5—H5B	109.6	O47—C47—C41	118.8 (3)
C6—C5—H5B	109.6	N4—C47—C41	117.8 (3)
H5A—C5—H5B	108.1	C42—C41—C46	115.6 (2)
N1—C6—C5	109.5 (2)	C42—C41—C47	122.4 (2)
N1—C6—H6A	109.8	C46—C41—C47	121.6 (3)
C5—C6—H6A	109.8	F42—C42—C43	119.5 (3)
N1—C6—H6B	109.8	F42—C42—C41	116.8 (2)
C5—C6—H6B	109.8	C43—C42—C41	123.7 (3)
H6A—C6—H6B	108.2	C42—C43—C44	118.0 (3)
N1—C11—C15	111.4 (2)	C42—C43—H43	121.0
N1—C11—H11A	109.3	C44—C43—H43	121.0
C15—C11—H11A	109.3	C43—C44—C45	121.2 (3)
N1—C11—H11B	109.3	C43—C44—H44	119.4
C15—C11—H11B	109.3	C45—C44—H44	119.4
H11A—C11—H11B	108.0	C46—C45—C44	118.1 (3)
C7A—O11—C12	105.7 (2)	C46—C45—H45	120.9
O13—C12—O11	108.4 (3)	C44—C45—H45	120.9
O13—C12—H12A	110.0	F46—C46—C45	119.2 (3)
O11—C12—H12A	110.0	F46—C46—C41	117.4 (2)
O13—C12—H12B	110.0	C45—C46—C41	123.3 (3)
O11—C12—H12B	110.0		
			
C6—N1—C2—C3	−63.5 (3)	C12—O11—C7A—C3A	4.4 (4)
C11—N1—C2—C3	173.9 (2)	C14—C3A—C7A—C17	0.1 (5)
N1—C2—C3—N4	54.5 (3)	O13—C3A—C7A—C17	−178.7 (3)
C2—C3—N4—C47	142.2 (3)	C14—C3A—C7A—O11	178.5 (3)
C2—C3—N4—C5	−48.1 (3)	O13—C3A—C7A—O11	−0.4 (4)
C47—N4—C5—C6	−140.1 (3)	C5—N4—C47—O47	3.6 (4)
C3—N4—C5—C6	49.5 (3)	C3—N4—C47—O47	173.0 (3)
C2—N1—C6—C5	65.0 (3)	C5—N4—C47—C41	−175.7 (2)
C11—N1—C6—C5	−172.1 (2)	C3—N4—C47—C41	−6.3 (4)
N4—C5—C6—N1	−57.3 (3)	O47—C47—C41—C42	−95.9 (4)
C6—N1—C11—C15	62.1 (3)	N4—C47—C41—C42	83.4 (4)
C2—N1—C11—C15	−177.2 (2)	O47—C47—C41—C46	76.3 (4)
C7A—O11—C12—O13	−6.7 (4)	N4—C47—C41—C46	−104.4 (4)
O11—C12—O13—C3A	6.5 (4)	C46—C41—C42—F42	179.2 (3)
C12—O13—C3A—C14	177.4 (3)	C47—C41—C42—F42	−8.2 (5)
C12—O13—C3A—C7A	−3.9 (4)	C46—C41—C42—C43	−0.2 (6)
O13—C3A—C14—C15	179.0 (3)	C47—C41—C42—C43	172.4 (4)
C7A—C3A—C14—C15	0.4 (5)	F42—C42—C43—C44	−179.1 (4)
C3A—C14—C15—C16	−0.8 (4)	C41—C42—C43—C44	0.3 (7)
C3A—C14—C15—C11	−177.6 (3)	C42—C43—C44—C45	−0.3 (7)
N1—C11—C15—C16	−107.8 (3)	C43—C44—C45—C46	0.3 (6)
N1—C11—C15—C14	69.0 (4)	C44—C45—C46—F46	179.4 (3)
C14—C15—C16—C17	0.8 (5)	C44—C45—C46—C41	−0.3 (5)
C11—C15—C16—C17	177.6 (3)	C42—C41—C46—F46	−179.5 (3)
C15—C16—C17—C7A	−0.3 (5)	C47—C41—C46—F46	7.8 (5)
C16—C17—C7A—O11	−178.2 (3)	C42—C41—C46—C45	0.3 (5)
C16—C17—C7A—C3A	−0.2 (5)	C47—C41—C46—C45	−172.4 (3)
C12—O11—C7A—C17	−177.4 (4)		

### 1-[(1,3-Benzodioxol-5-yl)methyl]-4-(2,6-difluorobenzoyl)piperazine (II) Hydrogen-bond geometry (Å, º)

**Table:**

*D*—H···*A*	*D*—H	H···*A*	*D*···*A*	*D*—H···*A*
C45—H45···O47^i^	0.95	2.58	3.204 (4)	123

Symmetry code: (i) −*x*+1, −*y*+2, *z*−1/2.

### 1-[(1,3-Benzodioxol-5-yl)methyl]-4-(2,4-dichlorobenzoyl)piperazine (III) Crystal data

**Table:**

C_19_H_18_Cl_2_N_2_O_3_	*F*(000) = 816
*M**_r_* = 393.25	*D*_x_ = 1.442 Mg m^−^^3^
Monoclinic, *P*2_1_/*n*	Mo *K*α radiation, λ = 0.71073 Å
*a* = 12.2889 (14) Å	Cell parameters from 4054 reflections
*b* = 12.3034 (14) Å	θ = 2.4–27.4°
*c* = 13.3667 (15) Å	µ = 0.38 mm^−^^1^
β = 116.295 (1)°	*T* = 173 K
*V* = 1811.9 (4) Å^3^	Block, colourless
*Z* = 4	0.49 × 0.48 × 0.38 mm

### 1-[(1,3-Benzodioxol-5-yl)methyl]-4-(2,4-dichlorobenzoyl)piperazine (III) Data collection

**Table:**

Bruker APEXII CCD diffractometer	4054 independent reflections
Radiation source: fine focus sealed tube	3545 reflections with *I* > 2σ(*I*)
Graphite monochromator	*R*_int_ = 0.017
Detector resolution: 0.3333 pixels mm^-1^	θ_max_ = 27.4°, θ_min_ = 2.4°
φ and ω scans	*h* = −15→11
Absorption correction: multi-scan (SADABS; Bruker, 2015)	*k* = −15→15
*T*_min_ = 0.776, *T*_max_ = 0.867	*l* = −17→17
9718 measured reflections	

### 1-[(1,3-Benzodioxol-5-yl)methyl]-4-(2,4-dichlorobenzoyl)piperazine (III) Refinement

**Table:**

Refinement on *F*^2^	0 restraints
Least-squares matrix: full	Hydrogen site location: inferred from neighbouring sites
*R*[*F*^2^ > 2σ(*F*^2^)] = 0.031	H-atom parameters constrained
*wR*(*F*^2^) = 0.087	*w* = 1/[σ^2^(*F*_o_^2^) + (0.043*P*)^2^ + 0.6034*P*] where *P* = (*F*_o_^2^ + 2*F*_c_^2^)/3
*S* = 1.04	(Δ/σ)_max_ = 0.001
4054 reflections	Δρ_max_ = 0.37 e Å^−^^3^
235 parameters	Δρ_min_ = −0.37 e Å^−^^3^

### 1-[(1,3-Benzodioxol-5-yl)methyl]-4-(2,4-dichlorobenzoyl)piperazine (III) Special details

**Table:**

Geometry. All esds (except the esd in the dihedral angle between two l.s. planes) are estimated using the full covariance matrix. The cell esds are taken into account individually in the estimation of esds in distances, angles and torsion angles; correlations between esds in cell parameters are only used when they are defined by crystal symmetry. An approximate (isotropic) treatment of cell esds is used for estimating esds involving l.s. planes.

### 1-[(1,3-Benzodioxol-5-yl)methyl]-4-(2,4-dichlorobenzoyl)piperazine (III) Fractional atomic coordinates and isotropic or equivalent isotropic displacement parameters (Å^2^)

**Table:**

	*x*	*y*	*z*	*U*_iso_*/*U*_eq_	
N1	0.52986 (10)	0.18706 (10)	0.64831 (10)	0.0293 (3)	
C2	0.48214 (12)	0.13857 (13)	0.72047 (12)	0.0335 (3)	
H2A	0.5451	0.1407	0.7989	0.040*	
H2B	0.4609	0.0616	0.6993	0.040*	
C3	0.37079 (12)	0.19934 (13)	0.71033 (11)	0.0321 (3)	
H3A	0.3360	0.1622	0.7553	0.038*	
H3B	0.3939	0.2740	0.7398	0.038*	
N4	0.27950 (10)	0.20437 (11)	0.59349 (9)	0.0310 (3)	
C5	0.32597 (13)	0.24338 (15)	0.51615 (12)	0.0376 (3)	
H5A	0.3457	0.3217	0.5292	0.045*	
H5B	0.2629	0.2343	0.4384	0.045*	
C6	0.43884 (13)	0.18029 (13)	0.53272 (12)	0.0342 (3)	
H6A	0.4173	0.1032	0.5122	0.041*	
H6B	0.4723	0.2103	0.4834	0.041*	
C11	0.64509 (13)	0.13691 (12)	0.66549 (15)	0.0371 (3)	
H11A	0.6295	0.0625	0.6341	0.045*	
H11B	0.6988	0.1313	0.7465	0.045*	
O11	0.87114 (9)	0.40074 (9)	0.46855 (9)	0.0362 (2)	
C12	0.83867 (16)	0.50226 (13)	0.49898 (14)	0.0407 (4)	
H12A	0.7743	0.5375	0.4326	0.049*	
H12B	0.9100	0.5511	0.5296	0.049*	
O13	0.79616 (10)	0.48304 (9)	0.58078 (10)	0.0391 (3)	
C3A	0.77539 (12)	0.37264 (11)	0.57687 (12)	0.0280 (3)	
C14	0.71953 (12)	0.31497 (12)	0.62874 (12)	0.0312 (3)	
H14	0.6897	0.3498	0.6751	0.037*	
C15	0.70819 (12)	0.20229 (12)	0.61057 (12)	0.0299 (3)	
C16	0.75523 (12)	0.15362 (12)	0.54436 (12)	0.0316 (3)	
H16	0.7471	0.0773	0.5330	0.038*	
C17	0.81427 (12)	0.21356 (12)	0.49382 (11)	0.0311 (3)	
H17	0.8479	0.1795	0.4501	0.037*	
C7A	0.82108 (11)	0.32314 (12)	0.51047 (11)	0.0269 (3)	
C47	0.15968 (12)	0.18798 (11)	0.55740 (11)	0.0273 (3)	
O47	0.08510 (9)	0.19648 (11)	0.45924 (8)	0.0426 (3)	
C41	0.11656 (11)	0.15520 (11)	0.64264 (10)	0.0240 (3)	
C42	0.09914 (11)	0.22960 (10)	0.71238 (10)	0.0236 (3)	
Cl42	0.13921 (3)	0.36508 (3)	0.71225 (3)	0.03486 (11)	
C43	0.04921 (11)	0.19988 (11)	0.78313 (11)	0.0261 (3)	
H43	0.0384	0.2518	0.8306	0.031*	
C44	0.01549 (11)	0.09273 (12)	0.78272 (11)	0.0276 (3)	
Cl44	−0.04771 (4)	0.05479 (4)	0.87079 (3)	0.04291 (12)	
C45	0.02990 (12)	0.01613 (11)	0.71408 (12)	0.0310 (3)	
H45	0.0052	−0.0570	0.7143	0.037*	
C46	0.08095 (12)	0.04794 (11)	0.64495 (12)	0.0291 (3)	
H46	0.0920	−0.0044	0.5980	0.035*	

### 1-[(1,3-Benzodioxol-5-yl)methyl]-4-(2,4-dichlorobenzoyl)piperazine (III) Atomic displacement parameters (Å^2^)

**Table:**

	*U*^11^	*U*^22^	*U*^33^	*U*^12^	*U*^13^	*U*^23^
N1	0.0239 (5)	0.0337 (6)	0.0344 (6)	0.0016 (5)	0.0165 (5)	0.0087 (5)
C2	0.0262 (6)	0.0420 (8)	0.0328 (7)	−0.0015 (6)	0.0135 (6)	0.0125 (6)
C3	0.0253 (6)	0.0480 (9)	0.0241 (6)	−0.0042 (6)	0.0119 (5)	0.0031 (6)
N4	0.0243 (5)	0.0478 (7)	0.0239 (5)	0.0012 (5)	0.0135 (5)	0.0061 (5)
C5	0.0304 (7)	0.0575 (10)	0.0312 (7)	0.0080 (7)	0.0194 (6)	0.0154 (7)
C6	0.0313 (7)	0.0454 (9)	0.0332 (7)	0.0022 (6)	0.0208 (6)	0.0042 (6)
C11	0.0294 (7)	0.0333 (8)	0.0526 (9)	0.0061 (6)	0.0217 (7)	0.0129 (7)
O11	0.0389 (6)	0.0424 (6)	0.0378 (6)	−0.0031 (5)	0.0265 (5)	0.0000 (5)
C12	0.0499 (9)	0.0396 (8)	0.0452 (9)	−0.0087 (7)	0.0326 (8)	−0.0021 (7)
O13	0.0537 (6)	0.0300 (5)	0.0509 (6)	−0.0066 (5)	0.0388 (6)	−0.0041 (5)
C3A	0.0256 (6)	0.0292 (7)	0.0320 (7)	0.0002 (5)	0.0153 (6)	−0.0009 (5)
C14	0.0307 (7)	0.0328 (7)	0.0389 (8)	0.0036 (6)	0.0235 (6)	0.0016 (6)
C15	0.0214 (6)	0.0319 (7)	0.0377 (7)	0.0051 (5)	0.0143 (6)	0.0062 (6)
C16	0.0250 (6)	0.0287 (7)	0.0378 (8)	0.0046 (5)	0.0110 (6)	−0.0008 (6)
C17	0.0255 (6)	0.0386 (8)	0.0291 (7)	0.0053 (6)	0.0120 (5)	−0.0041 (6)
C7A	0.0193 (6)	0.0379 (7)	0.0241 (6)	−0.0003 (5)	0.0103 (5)	0.0004 (5)
C47	0.0259 (6)	0.0336 (7)	0.0251 (6)	0.0026 (5)	0.0137 (5)	−0.0010 (5)
O47	0.0286 (5)	0.0743 (8)	0.0238 (5)	0.0021 (5)	0.0106 (4)	0.0031 (5)
C41	0.0186 (5)	0.0306 (7)	0.0226 (6)	0.0011 (5)	0.0090 (5)	−0.0002 (5)
C42	0.0232 (6)	0.0246 (6)	0.0233 (6)	−0.0024 (5)	0.0105 (5)	0.0000 (5)
Cl42	0.0493 (2)	0.02548 (18)	0.03616 (19)	−0.00753 (14)	0.02471 (17)	−0.00147 (13)
C43	0.0246 (6)	0.0315 (7)	0.0237 (6)	−0.0015 (5)	0.0121 (5)	−0.0013 (5)
C44	0.0211 (6)	0.0351 (7)	0.0261 (6)	−0.0021 (5)	0.0101 (5)	0.0063 (5)
Cl44	0.0431 (2)	0.0533 (2)	0.0397 (2)	−0.01166 (17)	0.02503 (17)	0.00825 (17)
C45	0.0276 (7)	0.0253 (7)	0.0361 (7)	−0.0013 (5)	0.0105 (6)	0.0046 (6)
C46	0.0274 (6)	0.0271 (7)	0.0311 (7)	0.0039 (5)	0.0115 (5)	−0.0025 (5)

### 1-[(1,3-Benzodioxol-5-yl)methyl]-4-(2,4-dichlorobenzoyl)piperazine (III) Geometric parameters (Å, º)

**Table:**

N1—C6	1.4540 (19)	O13—C3A	1.3787 (17)
N1—C2	1.4600 (17)	C3A—C14	1.3701 (19)
N1—C11	1.4665 (17)	C3A—C7A	1.3839 (19)
C2—C3	1.512 (2)	C14—C15	1.404 (2)
C2—H2A	0.9900	C14—H14	0.9500
C2—H2B	0.9900	C15—C16	1.389 (2)
C3—N4	1.4660 (17)	C16—C17	1.400 (2)
C3—H3A	0.9900	C16—H16	0.9500
C3—H3B	0.9900	C17—C7A	1.363 (2)
N4—C47	1.3462 (17)	C17—H17	0.9500
N4—C5	1.4658 (17)	C47—O47	1.2276 (17)
C5—C6	1.518 (2)	C47—C41	1.5088 (17)
C5—H5A	0.9900	C41—C42	1.3890 (18)
C5—H5B	0.9900	C41—C46	1.3950 (19)
C6—H6A	0.9900	C42—C43	1.3852 (17)
C6—H6B	0.9900	C42—Cl42	1.7383 (13)
C11—C15	1.5138 (19)	C43—C44	1.381 (2)
C11—H11A	0.9900	C43—H43	0.9500
C11—H11B	0.9900	C44—C45	1.380 (2)
O11—C7A	1.3817 (17)	C44—Cl44	1.7370 (13)
O11—C12	1.4242 (19)	C45—C46	1.384 (2)
C12—O13	1.4249 (17)	C45—H45	0.9500
C12—H12A	0.9900	C46—H46	0.9500
C12—H12B	0.9900		
			
C6—N1—C2	109.49 (11)	H12A—C12—H12B	108.4
C6—N1—C11	112.16 (12)	C3A—O13—C12	105.01 (11)
C2—N1—C11	111.54 (11)	C14—C3A—O13	128.09 (13)
N1—C2—C3	110.56 (11)	C14—C3A—C7A	122.22 (13)
N1—C2—H2A	109.5	O13—C3A—C7A	109.69 (12)
C3—C2—H2A	109.5	C3A—C14—C15	117.23 (13)
N1—C2—H2B	109.5	C3A—C14—H14	121.4
C3—C2—H2B	109.5	C15—C14—H14	121.4
H2A—C2—H2B	108.1	C16—C15—C14	119.91 (13)
N4—C3—C2	110.58 (12)	C16—C15—C11	121.81 (13)
N4—C3—H3A	109.5	C14—C15—C11	118.28 (13)
C2—C3—H3A	109.5	C15—C16—C17	122.09 (14)
N4—C3—H3B	109.5	C15—C16—H16	119.0
C2—C3—H3B	109.5	C17—C16—H16	119.0
H3A—C3—H3B	108.1	C7A—C17—C16	116.72 (13)
C47—N4—C5	120.16 (11)	C7A—C17—H17	121.6
C47—N4—C3	125.09 (11)	C16—C17—H17	121.6
C5—N4—C3	114.32 (11)	C17—C7A—O11	128.57 (12)
N4—C5—C6	110.25 (12)	C17—C7A—C3A	121.80 (13)
N4—C5—H5A	109.6	O11—C7A—C3A	109.63 (12)
C6—C5—H5A	109.6	O47—C47—N4	123.39 (13)
N4—C5—H5B	109.6	O47—C47—C41	118.97 (12)
C6—C5—H5B	109.6	N4—C47—C41	117.62 (11)
H5A—C5—H5B	108.1	C42—C41—C46	117.74 (12)
N1—C6—C5	110.42 (12)	C42—C41—C47	122.74 (12)
N1—C6—H6A	109.6	C46—C41—C47	119.22 (12)
C5—C6—H6A	109.6	C43—C42—C41	122.06 (12)
N1—C6—H6B	109.6	C43—C42—Cl42	117.72 (10)
C5—C6—H6B	109.6	C41—C42—Cl42	120.22 (10)
H6A—C6—H6B	108.1	C44—C43—C42	118.17 (12)
N1—C11—C15	111.53 (11)	C44—C43—H43	120.9
N1—C11—H11A	109.3	C42—C43—H43	120.9
C15—C11—H11A	109.3	C45—C44—C43	121.85 (12)
N1—C11—H11B	109.3	C45—C44—Cl44	119.64 (11)
C15—C11—H11B	109.3	C43—C44—Cl44	118.50 (11)
H11A—C11—H11B	108.0	C44—C45—C46	118.71 (13)
C7A—O11—C12	105.00 (10)	C44—C45—H45	120.6
O11—C12—O13	108.54 (12)	C46—C45—H45	120.6
O11—C12—H12A	110.0	C45—C46—C41	121.47 (13)
O13—C12—H12A	110.0	C45—C46—H46	119.3
O11—C12—H12B	110.0	C41—C46—H46	119.3
O13—C12—H12B	110.0		
			
C6—N1—C2—C3	−61.31 (16)	C12—O11—C7A—C17	172.09 (14)
C11—N1—C2—C3	173.95 (13)	C12—O11—C7A—C3A	−8.17 (15)
N1—C2—C3—N4	55.00 (16)	C14—C3A—C7A—C17	−0.7 (2)
C2—C3—N4—C47	136.85 (14)	O13—C3A—C7A—C17	178.96 (13)
C2—C3—N4—C5	−50.77 (17)	C14—C3A—C7A—O11	179.50 (12)
C47—N4—C5—C6	−136.22 (14)	O13—C3A—C7A—O11	−0.81 (15)
C3—N4—C5—C6	50.99 (18)	C5—N4—C47—O47	5.4 (2)
C2—N1—C6—C5	61.64 (16)	C3—N4—C47—O47	177.41 (14)
C11—N1—C6—C5	−173.97 (12)	C5—N4—C47—C41	−175.89 (13)
N4—C5—C6—N1	−55.75 (17)	C3—N4—C47—C41	−3.9 (2)
C6—N1—C11—C15	69.26 (16)	O47—C47—C41—C42	−100.59 (16)
C2—N1—C11—C15	−167.51 (13)	N4—C47—C41—C42	80.68 (17)
C7A—O11—C12—O13	14.03 (16)	O47—C47—C41—C46	72.85 (18)
O11—C12—O13—C3A	−14.51 (16)	N4—C47—C41—C46	−105.88 (15)
C12—O13—C3A—C14	−170.92 (15)	C46—C41—C42—C43	0.52 (19)
C12—O13—C3A—C7A	9.41 (16)	C47—C41—C42—C43	174.06 (12)
O13—C3A—C14—C15	179.33 (14)	C46—C41—C42—Cl42	−178.99 (10)
C7A—C3A—C14—C15	−1.0 (2)	C47—C41—C42—Cl42	−5.46 (17)
C3A—C14—C15—C16	1.5 (2)	C41—C42—C43—C44	−0.42 (19)
C3A—C14—C15—C11	−179.07 (12)	Cl42—C42—C43—C44	179.11 (10)
N1—C11—C15—C16	−132.47 (14)	C42—C43—C44—C45	−0.3 (2)
N1—C11—C15—C14	48.08 (19)	C42—C43—C44—Cl44	−179.77 (10)
C14—C15—C16—C17	−0.2 (2)	C43—C44—C45—C46	0.8 (2)
C11—C15—C16—C17	−179.64 (13)	Cl44—C44—C45—C46	−179.70 (10)
C15—C16—C17—C7A	−1.5 (2)	C44—C45—C46—C41	−0.7 (2)
C16—C17—C7A—O11	−178.31 (12)	C42—C41—C46—C45	0.03 (19)
C16—C17—C7A—C3A	2.0 (2)	C47—C41—C46—C45	−173.74 (12)
